# A Rare Case of Successfully Treated Double Valve Infective Endocarditis Caused by *Pseudomonas aeruginosa*

**DOI:** 10.3390/ijms231911127

**Published:** 2022-09-22

**Authors:** Raluca Tomoaia, Alexandru Oprea, Irina Sandu, Vlad Danu, Dana Pop, Dumitru Zdrenghea, Alexandra Dădârlat-Pop, Ioan Alexandru Minciună, Ioana Maria Chețan, Nicoleta Cosmina Hada, Ruxandra Ștefana Beyer

**Affiliations:** 1 Cardiology Department, Heart Institute “N. Stăncioiu”, 400001 Cluj-Napoca, Romania; 2 5th Department of Internal Medicine, Faculty of Medicine, “Iuliu Hațieganu” University of Medicine and Pharmacy, 400012 Cluj-Napoca, Romania; 3 Cardiology Department, Clinical Rehabilitation Hospital, 400347 Cluj-Napoca, Romania; 4 Cardiovascular Surgery Department, Heart Institute “N. Stăncioiu”, 400001 Cluj-Napoca, Romania; 5 7th Department of Surgery, Faculty of Medicine, “Iuliu Hațieganu” University of Medicine and Pharmacy, 400012 Cluj-Napoca, Romania; 6 Radiology Department, Heart Institute “N. Stăncioiu”, 400001 Cluj-Napoca, Romania; 7 Infectious Disease Department, Heart Institute “N. Stăncioiu”, 400001 Cluj-Napoca, Romania

**Keywords:** infective endocarditis, *Pseudomonas aeruginosa*, multimodality imaging, ghost catheter fibrin sleeve

## Abstract

*Pseudomonas aeruginosa* is a rare yet particularly aggressive infective endocarditis pathogen. We describe a case of successfully managed double-valve *P. aeruginosa* infective endocarditis, in which the presumed source of bacteremia was a long-term tunneled central venous catheter used for hemodialysis.

## 1. Introduction

Fibrin sheath formation around an indwelling central venous catheter is common and usually uncomplicated. The persistence of the fibrin sheath after catheter removal, also known as a “ghost” catheter fibrin sleeve (GCFS), occurs in only 15% of patients and is usually an incidental finding [1,2,3].

*Pseudomonas aeruginosa* is a rare infective endocarditis (IE) pathogen, accounting for approximately 3% of all cases [2]. The majority of reports indicate that *P. aeruginosa* IE is more aggressive and has a higher mortality rate [4]. Ninety percent of these cases are associated with intravenous drug use (IDU) [1]. Consequently, the majority of patients present with right-sided valve IE. More cases of right-sided *P. aeruginosa IE* have recently been reported as connected with healthcare infections, owing to an increase in the number of invasive medical procedures and intravascular device insertion. Although the incidence of *P. aeruginosa* IE remains significantly low and the clinical characteristics of these patients are not well known, data from the literature show that right-sided IE is associated with more embolic events than left-sided IE. Thus, the most commonly reported consequences of right-sided *P. aeruginosa* IE include valvular regurgitations, embolic events and abscess formation in the lungs [5].

Left sided *P. aeruginosa* IE is uncommon, with only a few cases reported in literature, and is occasionally caused by nosocomial infections. According to one study, left-sided IE accounts for only 0.6% of all IE cases [6].

However, *P. aeruginosa* IE affecting both the right and the left valves has not yet been reported. We describe the management and outcome of a hemodialysis patient who developed tricuspid and aortic valve IE as a result of catheter-related *P. aeruginosa* bacteremia.

## 2. Case Report

A 58-year-old male with a past medical history of tuberculosis treated with antibiotics (2010) and end-stage renal disease presented to the Cardiology Department for progressive generalized malaise and intermittent fever. The patient was diagnosed with severe renal failure caused by Leptospirosis in 2020 and began maintenance hemodialysis using a right internal jugular tunneled catheter. He had an arteriovenous fistula placed nine months later. The central venous catheter (CVC) was used for dialysis until the arteriovenous fistula maturated, at which point it was removed, one year after initial placement.

The patient developed cough, fever, and fatigue one week after the extraction, at which time he was examined at another clinic, but cultures tested from the central line were negative. The chest X-ray, on the other hand, revealed two left-lung lobar pulmonary opacities, suggestive of pneumonia. Despite receiving initial one week of therapy with cefuroxime for suspected community-acquired pneumonia, he continued to be febrile. Blood cultures were found to be positive for *P. aeruginosa*. At that time, echocardiography revealed IE of the tricuspid valve, with one vegetation having a length of 10 mm. As a result, antibiotic therapy was started with ceftazidime and gentamycin followed by meropenem alone and was continued for a total of 42 days. Considering the absence of inflammatory syndrome and of evidence of IE on the control echocardiography, the patient was discharged after six weeks.

One month later, the patient was admitted to our Cardiology Department due to symptoms deteriorating. On examination, he was febrile to 37.7 °C, with severe asthenia and disorientation, as well as a pale grey facies and peripheral oedema. The patient had a new diastolic murmur that was suggestive of aortic regurgitation. There were no notable changes on the electrocardiogram.

Laboratory testing revealed a severe inflammatory syndrome, with a C-reactive protein level of 233 mg/L, leukocytosis of 12,000/μL, and neutrofilia. Severe anemia (hemoglobin of 6.8 g/dL) and thrombocytopenia (21,000/μL) were also present. NT-proBNP levels were increased, reaching a value of 35,000 pg/mL. The renal function was severely impaired, as expected given the patient’s long-term hemodialysis, with a creatinine level of 9 mg/dL and a BUN level of 230 mg/dL.

**Figure 1:**
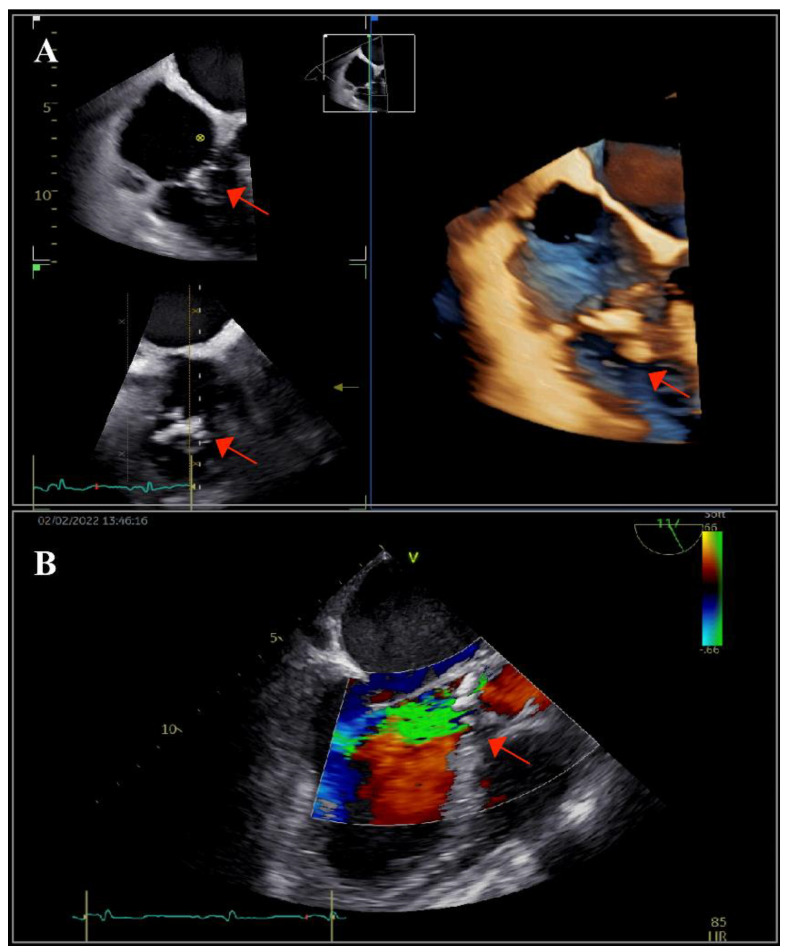
Transesophageal echocardiography: (**A**) bi- and tri-dimensional acquisitions depicting multiple vegetations on the tricuspid valve (arrow) and (**B**) long-axis view showing the aortic valve with vegetations (arrow) and the severe aortic regurgitation. The left aortic coronary cusp was perforated, which was later confirmed during surgery.

**Figure 2:**
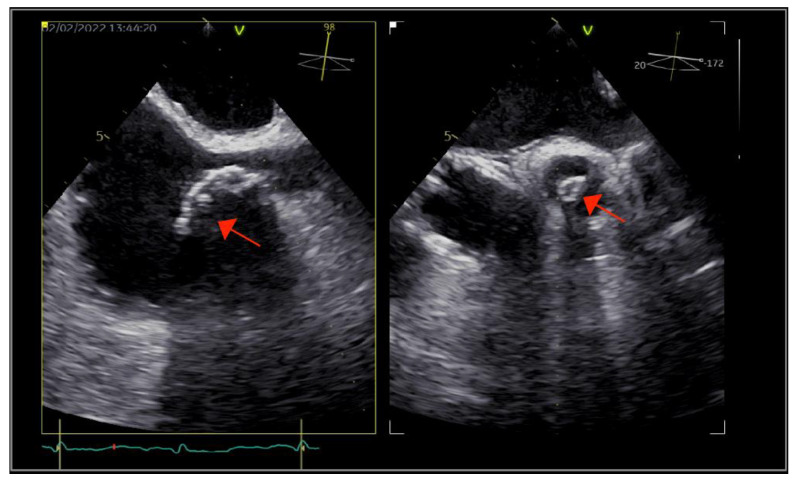
Multi-dimensional transesophageal echocardiography acquisitions showing an inhomogeneous calcified mass at the level of the superior vena cava, with right atrial protrusion (arrow).

The patient underwent transthoracic echocardiography, which revealed severe aortic and tricuspid regurgitation, moderately reduced left ventricular ejection fraction (35%) and preserved right ventricular systolic function. There were multiple small mobile masses attached to the aortic valve, indicating vegetations. Because the tricuspid valve was thickened, tricuspid endocarditis was also suspected. These findings were corroborated by transesophageal echocardiography (TOE), which revealed multiple vegetations at the level of both the aortic and tricuspid valves that were better visualized with three-dimensional acquisitions (Figure 1). There were no paravalvular complications. Furthermore, TOE visualized an inhomogeneous mass at the level of the superior vena cava (SVC), prolapsing into the right atrium, indicating the possibility of a fibrin sheath subsequent to the removal of the CVC or thrombus (Figure 2).

At this stage, since the symptoms initially began shortly after removal of the central venous line, a persistent catheter-related infection was presumed. A Heart Team, consisting of a cardiologist, an infectious disease physician, a cardiac surgeon, and a general surgeon, was gathered. As blood cultures were positive for *P. aeruginosa*, antibiotic therapy with meropenem (2 × 500 mg/day for 42 days) and amikacin (500 mg post-hemodialysis for 21 days) was initiated based on the antibiotic susceptibility (meropenem MIC ≤ 0.25 and amikacin MIC ≤ 2, as stated in the antibiogram determined with the VITEK automatic method following the EUCAST standard). The patient’s inflammatory symptoms improved after receiving therapy and two red blood cell and three platelet transfusions. On day 10, blood cultures were negative again. In addition to the medical management, the heart team decided for valve replacement.

**Figure 3:**
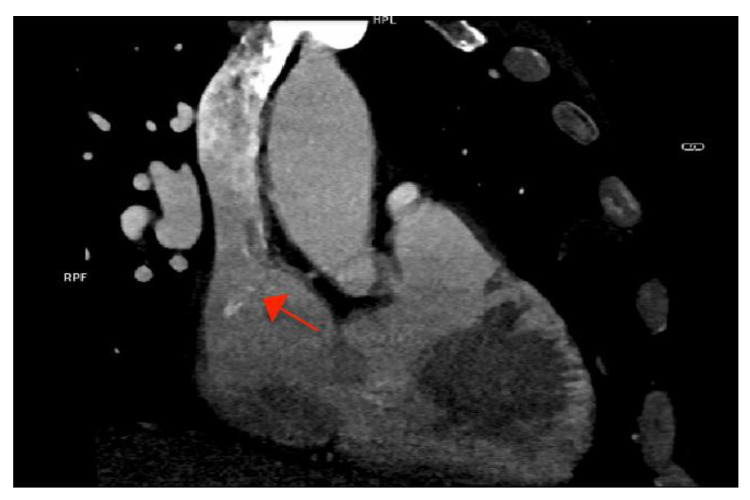
Cardiac computed tomography showing the calcified fibrin sheath in the superior vena cava (arrow), without evidence of vegetations.

As part of the imaging assessment approach in IE, a thoracic, abdominal, pelvic, and cerebral computed tomography (CT) scan were conducted. The GCFS was visible in the SVC, but there was no evidence of vegetation at this level (Figure 3).

**Figure 4:**
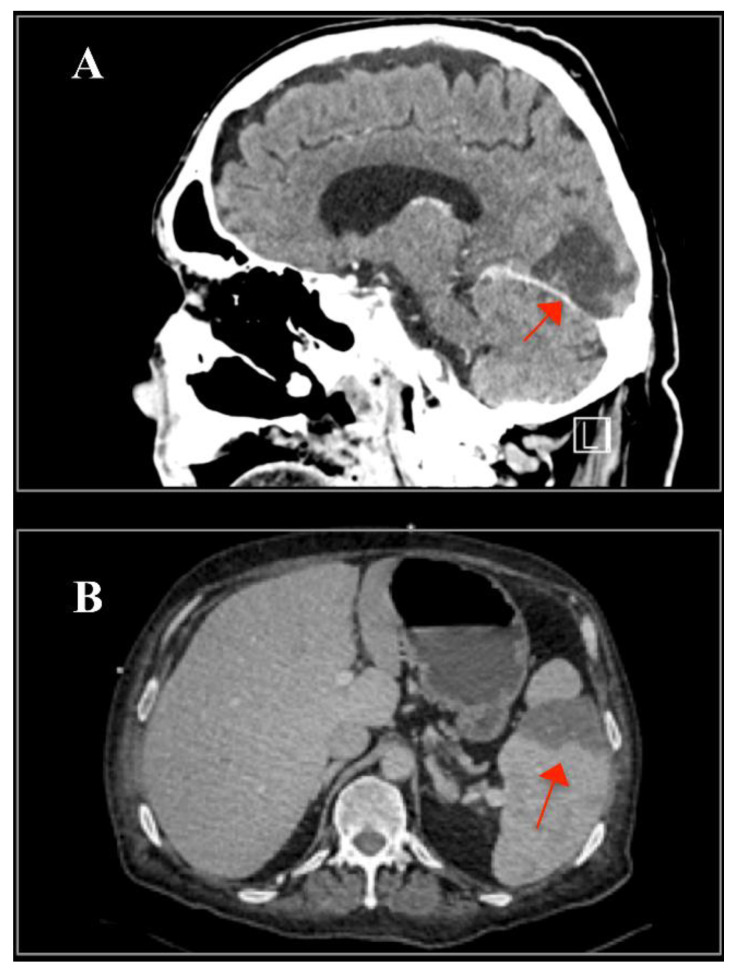
(**A**) Cerebral computed tomography (CT) demonstrating subacute right occipital stroke (arrow) and (**B**) abdominal CT showing one of the splenic infarctions (arrow).

There was a subacute right occipital stroke and splenomegaly with splenic infarctions, both of which were interpreted as embolic consequences of the IE (Figure 4).

**Figure 5:**
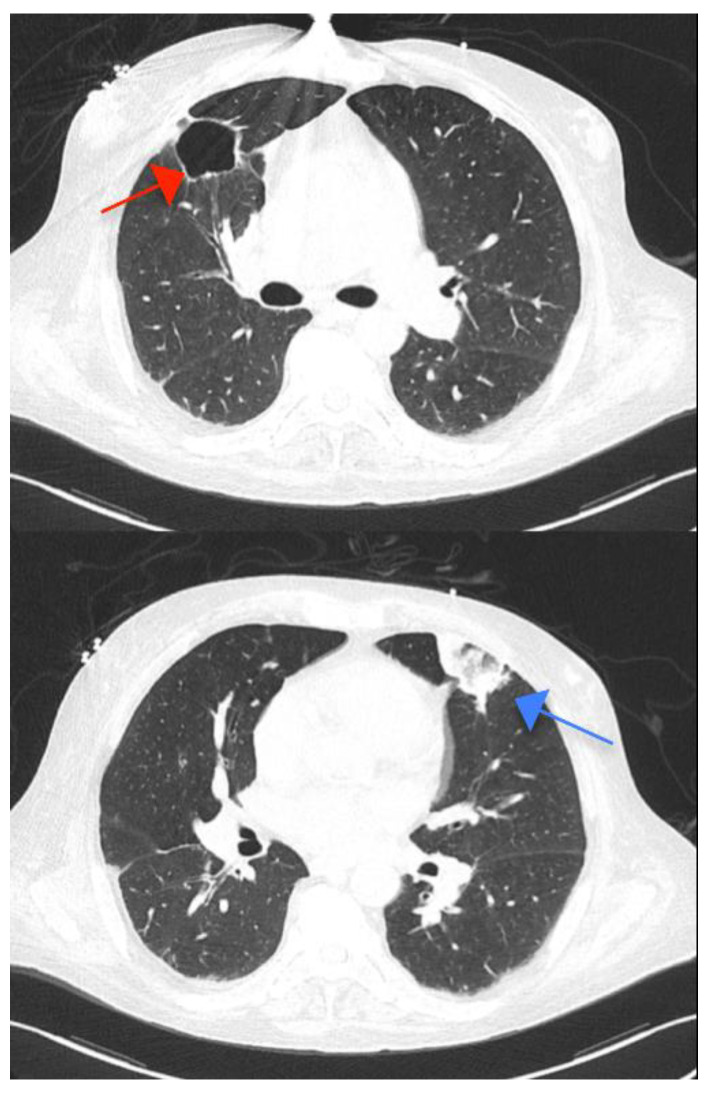
Thoracic computed tomography revealing one of the four cavities in the right upper lobe (red arrow) and one triangular consolidation in the left superior lobe, raising the suspicion of tuberculosis reactivation (blue arrow).

Four cavities in the lungs were identified as tuberculosis sequelae (Figure 5); however, two lobar consolidations raised the possibility of tuberculosis reactivation, which was later excluded by negative sputum smear, presumably indicating embolization from the right-sided IE. Additional coronary CT angiography showed no significant stenosis in the coronary arteries.

**Figure 6:**
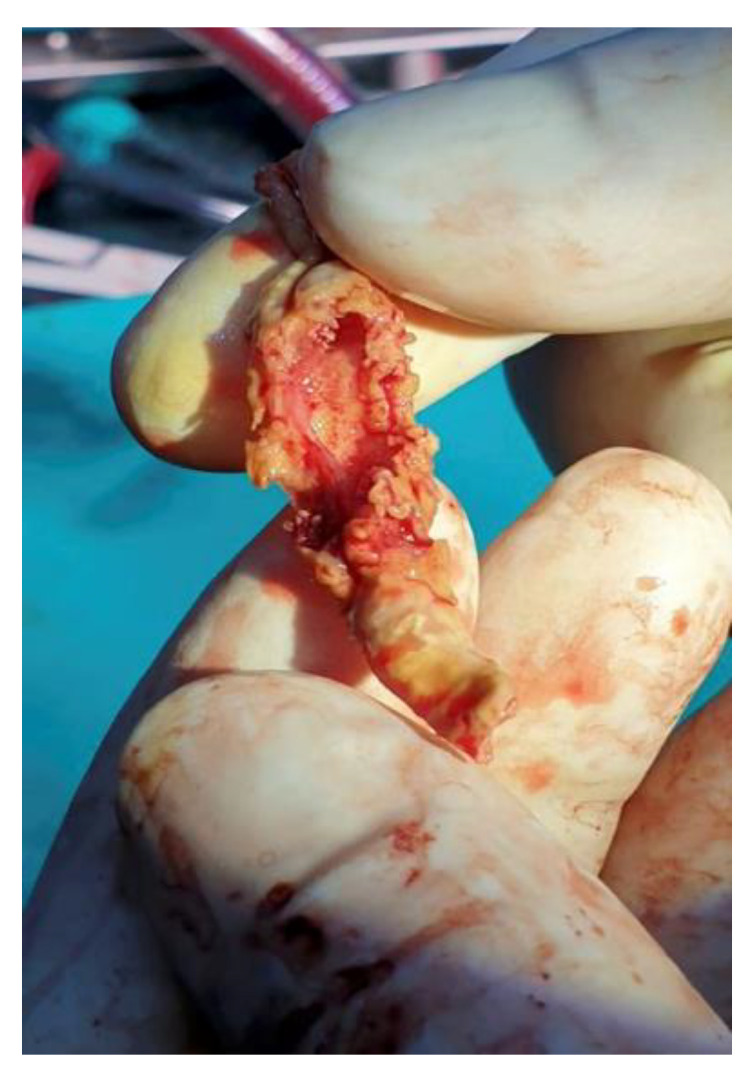
Operative view of the calcified fibrin sheath after extraction from the superior vena cava.

Following the exclusion of tuberculosis reactivation, the patient underwent cardiac surgery on day 16. Aortic Sorin Carbomedics no. 23 and tricuspid Sorin Carbomedics no. 31 prostheses were implanted. The mass in the SVC was also removed (Figure 6). Because there were no signs of splenic abscesses in the setting of splenomegaly with splenic infarctions, it was decided that urgent splenectomy was not necessary.

A subsequent biopsy revealed inflammatory infiltration at the level of the aortic valve. Interestingly, only thrombotic material, which was rich in fibrin but lacking in figurate components, as well as minor calcifications, were identified at the level of the SVC mass. The smear revealed no Gram-negative bacteria, and the cultures were negative. PCR was not performed from the excised valves due to lack of availability of the test in our country.

After surgery, within the first four postoperative hours, 900 mL of hemorrhagic effusion was drained from the pericardium. A diffuse hemorrhage in the posterior sternum was identified and cauterized in the absence of an active bleeding source. The patient’s short-term outcome after surgery was satisfactory, but on the seventh postoperative day, he contacted a COVID-19 positive patient and tested positive with the polymerase chain reaction (PCR) test. He developed cough and a temperature of 37.6 °C, thus molnupiravir 800 mg bid was administered for 5 days. Blood cultures in the setting of persistent fever were positive for methicillin-resistant *Staphylococus hominis*, indicating an infection due to the catheter used for medication administration. According to our hospital’s protocols, the catheter was removed and vancomycin was administered for a total of 14 days based on antibiotic susceptibility (vancomycin MIC = 1 as stated in the antibiogram determined with the VITEK automatic method following the EUCAST standard) with good clinical outcome. Repeated blood cultures at one week were negative and the patient was transferred to a territorial hospital for the continuation of antibiotics. Meropenem was continued until day 42, as recommended in the 2015 ESC Guidelines for the management of infective endocarditis as the minimum duration of treatment for Gram-negative bacteria [7], and was then discontinued due to good clinical, biological, and echocardiographic response. Amikacin was discontinued after 21 days due to ototoxicity concerns.

Transthoracic echocardiography was repeated one month following surgery and revealed normal-functioning prosthetic valves. At the three-month follow-up, the patient was still doing well clinically.

## 3. Discussion

We present a rare case of *P. aeruginosa* double-valve IE in a patient without prior history of IDU. When compared to other types of bacteria, *P. aeruginosa* is an infrequent cause of IE. Following IDU, healthcare exposure is the second most often cited risk factor. Because mortality for healthcare-associated *P. aeruginosa* IE was shown to be very high (40%) in a recently published review [4], early identification is critical for a successful outcome.

Antibiotic regimens for *P. aeruginosa* IE consist of six weeks of two intravenous anti- pseudomonal drugs from two synergistic antibacterial classes [8]. In gram-negative infections, the combination of beta-lactam and aminoglycoside is recommended. Medical treatment alone has previously been found to be effective in left-sided IE caused by *P. aeruginosa* [9,10,11]. However, therapy is inconsistent, recurrence is common, and surgery is frequently required [12]. In the case of right-sided valve IE, surgery is necessary if antibiotics fail, whereas in left-sided valve infections, early surgery is often required [6]. Aside from the uncommon etiological agent for IE, another notable feature of this clinical case was the infection of both a right and a left valve. To the best of our knowledge, this is the first report of a patient with numerous comorbidities and catheter-related double-valve *P. aeruginosa* IE who was effectively managed with combination antibiotic therapy and valve replacement.

Regarding the time between admissions, it appears that our patient had persistent bacteremia despite antibiotic therapy. *P. aeruginosa* is an uncommon and multiresistant bacterium that falls under the category of “uncontrolled infection” (infection caused by fungus or multiresistant organism with an indication for urgent/elective surgery) according to the 2015 ESC Guidelines for the management of infective endocarditis [7]. The initial antibiotic therapy was used before the patient was referred to our hospital. In our opinion, the initial therapy failed due to the lack of surgical consultation and intervention at that time, as well as likely owing to the meropenem monotherapy. Also, this case demonstrates that, in spite of the initial negative control echocardiogram, an earlier TOE may have allowed for a faster intervention prior to cerebral and splenic embolization.

Long-term CVC use is known to increase the risk of catheter-related infection. CVC infection can occur either exogenously (straight from the catheter or infusate exit site) or endogenously (through bacteremia). There are two hypotheses on the relationship between the fibrin sheath and bacterial adhesion: the fibrin sheath either hinders bacterial adhesion or interferes with the immune function, resulting in infection [13]. Another recent report described two cases of GCFS identified by TOE following the removal of a CVC in hemodialysis patients, both of whom had positive *Staphylococcus aureus* blood cultures [14]. Furthermore, a multicenter study previously revealed a link between CVC-related sepsis and CVC-related central vein thrombosis [15]. Sepsis was more common in patients with catheter-related central vein thrombosis than in those without, with a 2.6-fold increased risk. However, it remains unclear whether the infection is caused by CVC-related central venous thrombosis or if the thrombosis is caused by sepsis. In the case of this patient, who was found to have a GFSC with thrombotic material, it may be argued that the CVC may have induced bacteria into the bloodstream. However, because no other sources of infection were identified, it was presumed that the infection originated endogenously via the CVC.

## 4. Conclusions

In conclusion, *P. aeruginosa* IE unrelated to IDU is uncommon but particularly aggressive, due to early relapse. In a patient on hemodialysis, persistent *P. aeruginosa* bacteremia should raise the possibility of IE.

## Data Availability

Not applicable.
